# Impact of Transgenerational Nutrition on Nonalcoholic Fatty Liver Disease Development: Interplay between Gut Microbiota, Epigenetics and Immunity

**DOI:** 10.3390/nu16091388

**Published:** 2024-05-03

**Authors:** Hong-Tai Tzeng, Wei-Chia Lee

**Affiliations:** 1 Institute for Translational Research in Biomedicine, Kaohsiung Chang Gung Memorial Hospital, Kaohsiung 83301, Taiwan; htay11@cgmh.org.tw; 2 Division of Urology, Kaohsiung Chang Gung Memorial Hospital, Kaohsiung 83301, Taiwan; 3 College of Medicine, Chang Gung University, Taoyuan 33332, Taiwan

**Keywords:** nonalcoholic fatty liver disease, maternal diet, gut microbiota, epigenetics, immune response, transgeneration

## Abstract

Nonalcoholic fatty liver disease (NAFLD) has emerged as the most prevalent pediatric liver disorder, primarily attributed to dietary shifts in recent years. NAFLD is characterized by the accumulation of lipid species in hepatocytes, leading to liver inflammation that can progress to steatohepatitis, fibrosis, and cirrhosis. Risk factors contributing to NAFLD encompass genetic variations and metabolic disorders such as obesity, diabetes, and insulin resistance. Moreover, transgenerational influences, resulting in an imbalance of gut microbial composition, epigenetic modifications, and dysregulated hepatic immune responses in offspring, play a pivotal role in pediatric NAFLD development. Maternal nutrition shapes the profile of microbiota-derived metabolites in offspring, exerting significant influence on immune system regulation and the development of metabolic syndrome in offspring. In this review, we summarize recent evidence elucidating the intricate interplay between gut microbiota, epigenetics, and immunity in fetuses exposed to maternal nutrition, and its impact on the onset of NAFLD in offspring. Furthermore, potential therapeutic strategies targeting this network are also discussed.

## 1. Introduction

Nonalcoholic fatty liver disease (NAFLD) is a condition characterized by the accumulation of excess fat in the liver, unrelated to excessive alcohol consumption, and has become the most common pediatric liver disease in recent years [1]. It encompasses a spectrum of manifestations, including steatosis, nonalcoholic steatohepatitis (NASH), and NASH-induced cirrhosis, subsequently increasing the risk of hepatocellular carcinoma (HCC) [2]. Among chronic liver diseases, NAFLD is the most prevalent, affecting approximately 25% of the global population. The development of NAFLD is closely associated with obesity and an elevated body mass index (BMI). Moreover, more than half of individuals with type 2 diabetes mellitus have NAFLD, categorizing NAFLD as a component of metabolic syndrome [3,4]. Various risk factors, including genetics, epigenetics, and environmental stimuli such as gut microbiota and nutrition, contribute to dysregulated lipid metabolism in the liver and the initiation of liver inflammation [5]. Chronic liver inflammation in NAFLD promotes tissue remodeling and fibrogenesis, which can lead to the progression of liver fibrosis and cirrhosis. Ultimately, this progression increases the risk of developing NAFLD-related hepatocellular carcinoma (HCC).

**Figure 1:**
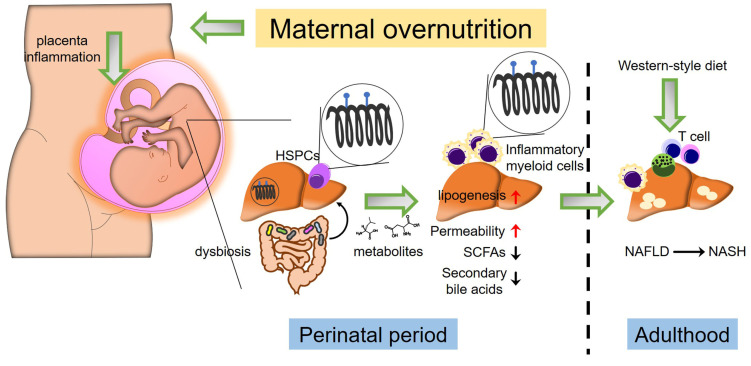
Cross-talk between gut microbiota, epigenetics, and immune response in maternal diet-induced offspring NAFLD. Maternal excessive nutrition uptake leading to obesity induces placental inflammation and alters fetal gut microbial colonization patterns. Fetal gut dysbiosis induced by abnormal inflammation resulting from maternal overnutrition (e.g., Western-style diet) increases gut intestinal permeability. Additionally, gut dysbiosis results in reduced short-chain fatty acids (SCFAs) and secondary bile acids. Dysbiosis and inflammatory conditions within the placenta induce alterations in epigenetics, such as methylation patterns in fetal liver hematopoietic stem/progenitor cells (HSPCs) and hepatic cells. Consequently, this leads to an increase in myeloid cells exhibiting inflammatory phenotypes and promotes lipogenesis in the fetal liver. These perinatal perturbations increase the risk of offspring NAFLD development in adulthood when exposed to another “hit” (e.g., Western-style diet).

The hypothesis of Developmental Origins of Health and Disease (DOHaD) suggests that intrauterine exposure to environmental stimuli, such as maternal malnutrition and maternal pathologies, influences the development and progression of diseases in offspring throughout adulthood [6]. Emerging evidence indicates that maternal nutritional factors play a significant role in shaping the metabolic health of offspring. Substances absorbed by the mother during gestation may have potential benefits in preventing the development of metabolic disorders in offspring. However, they can also increase the susceptibility to chronic metabolic syndromes, such as NAFLD [7]. Hence, comprehending the mechanisms of perinatal exposure to maternal nutrition, which may program NAFLD *in utero*, is crucial for identifying perinatal conditions and defining promising strategies for addressing this disease. Figure 1 summarizes the perinatal perturbations involved in fetal programming and the development of adulthood NAFLD (Figure 1). In this review, we outline the potential mechanisms involved in maternal nutritional regulation during the perinatal period, focusing on NAFLD risk factors such as gut microbiota, epigenetic regulation, and immunological responses, as well as the disease’s pathogenesis in adulthood.

## 2. Immune-Mediated Pathogenesis of Nonalcoholic Fatty Liver Disease (NAFLD)

Inflammation plays a critical role in the progression of NAFLD, especially at the onset of NASH. The accumulation of innate and adaptive immune cells in fatty liver contributes significantly to the pathogenesis of NASH [8]. Kupffer cells (KCs), the liver-resident macrophages, are pivotal innate immune cells in the development of NASH [9]. During the initial stages of NASH development, heightened hepatic LPS levels arise from increased intestinal permeability caused by gut dysbiosis induced by nutritional insults and fatty acids stimulate Kupffer cells (KCs) to secrete tumor necrosis factor α (TNFα) and interleukin (IL)-1β, initiating hepatic inflammation [10,11]. In addition to macrophages, another population of innate immune cells, dendritic cells (DCs), also play a role in the progression of NASH. Recently, type 1 conventional DCs have been shown to contribute to liver pathology in NASH by promoting inflammatory T cell reprogramming [12].

### 2.1. Development of NASH

It is observed that approximately 5–10% of individuals with NAFLD advance to NASH [13]. Within the context of NAFLD, insulin resistance emerges as a key factor in the development of NASH, given its critical role in establishing lipotoxicity, inducing oxidative stress, and facilitating the release of proinflammatory cytokines [14]. Moreover, the accumulation of free fatty acids (FFAs) flux in the liver raises the levels of hepatic triglycerides, free cholesterol, and other lipid metabolites, thereby promoting lipotoxicity and subsequent mitochondrial dysfunction accompanied by oxidative stress. Consequently, the heightened production of reactive oxygen species (ROS) and activation of endoplasmic reticulum (ER) stress-associated mechanisms, such as the unfolded protein response, contribute to a necrotic inflammatory response and the accumulation of liver damage. Furthermore, increased intestinal permeability resulting from circulating lipid metabolites amplifies the release of molecules that activate inflammation, such as lipopolysaccharides (LPS), thereby fostering hepatic production of proinflammatory cytokines [15]. Hence, factors influencing hepatic fat content and liver inflammatory responses collectively contribute to the progression of NASH.

#### 2.1.1. Role of CD8^+^ T Cells

Emerging evidence has implicated adaptive immune cells, including T and B cells, in the initiation and progression of NASH [16]. Among them, T cells play a crucial role in triggering hepatic inflammation. The expression of co-receptors on the surface of T cells classifies them into CD8^+^ and CD4^+^ subsets. Accumulating data show an increase in CD8^+^ T cells in the livers of NASH patients [17,18] and in murine models of the disease [19]. Furthermore, depletion of CD8^+^ T cells has been shown to ameliorate NASH development [20], suggesting that preventing the activation of CD8^+^ T cells suppresses disease progression. Consequently, the improvement in NASH observed in CD8^+^ T cell-deficient mice is associated with restored hepatic function and reduced liver damage [21].

#### 2.1.2. Activation of CD8^+^ T Cells during NASH

The hepatic recruitment of CD8^+^ T cells is mediated by KCs. During the development of NAFLD, increased lipid species and their metabolites activate KCs to produce ROS and proinflammatory cytokines, which in turn induces hepatocytes undergoing cell death. The injured hepatocytes thus secrete damage-associated molecular patterns (DAMPs) that can be recognized by pattern recognition receptors expressed in KCs and activate these cells to produce immune mediators to recruit CD8^+^ T cells [13]. Additionally, elevated hepatic LPS level due to NAFLD-associated disruption of intestinal barrier also activates KCs through engagement of Toll-like receptor [14]. Recently, activated KCs has been demonstrated to participate in the hepatic infiltration of CD8^+^ T cells in cooperatively with activated platelets [15]. Nevertheless, the cellular mechanisms of interplay between hepatic environment and immune reactions that attract CD8^+^ T cells into livers are needed further investigation. Activation of CD8^+^ T cells plays a pivotal role in the pathogenesis of HASH. In fact, a subpopulation of hepatic CD8^+^ T cells, characterized as CXCR6^+^PD1^+^CD8^+^ T cells, has been identified as an important player responsible for NASH phenotype [17]. By using a mouse model with key features of human NASH, Dudek et al. demonstrated that downregulation of transcription factor FOXO1 and increased expression of CXCR6 in liver-infiltrating CD8^+^ T cells was induced by IL-15. Moreover, the IL-15-dependent mechanism caused these cells susceptible to metabolic changes in the NASH microenvironment of mice fed with choline-deficient high-fat diet and Western diet regimen. Consequently, CXCR6^+^CD8^+^ T cells acquired autoaggressive character that killed hepatocytes by Fas/Fas ligand interaction and independent of MHC-I-restricted fashion. These findings indicate that CD8^+^ T cells influences the evolution of the hepatic disease through response to specific metabolic changes during NAFLD progression and NASH development.

#### 2.1.3. Role of CD4^+^ T Cells

CD4^+^ T cells represent another type of adaptive immune cell involved in regulating NASH development. Indeed, an accumulation of CD4^+^ T cells with an inflammatory phenotype is observed in the livers of NASH patients and in murine models of the disease [22]. Deficiency in CD4^+^ T cells in NASH mice reduces hepatic inflammatory cytokines and disease severity, indicating that NASH progression is dependent on CD4^+^ T cells. Moreover, in patients with NASH, the frequency of interferon (IFN)-γ-expressing CD4^+^ T cells is higher than that observed in healthy individuals [23]. Consistent with these findings, knockout of IFN-γ attenuates disease progression in mice, leading to reduced activation and hepatic infiltration of immune cells [24]. Collectively, these results suggest that immune activation regulation can exert control over the development and progression of NASH.

#### 2.1.4. Activation of CD4^+^ T Cells during NASH

The main CD4^+^ T cell population can be divided into Th1, Th2 and Th17 subsets. Among them, Th1 cells typically produce proinflammatory cytokines such as IFN-γ, IL-2 and TNF-α, and exhibit an increase in livers of patients with NASH and in mice model fed with high caloric diets [25,26]. The master transcriptional factor GATA-3 drives the differentiation of Th2 and promotes the production of IL-4, IL-5 and IL-13 [27]. In a mouse model of NAFLD, an increase ratio of Th1/Th2 in mesenteric lymph nodes was observed. Adoptive transfer of these CD4^+^ T cells into mice fed with high-fat diet exacerbated hepatic inflammation [28]. However, the role of these CD4^+^ T cell subsets in NASH pathogenesis still need to be further investigation. In contrast, IL-17-producing Th17 cells has been implicated in the pathogenesis of NASH. A recent study showed that mice fed with high-fat and high fructose diet exhibited an increased hepatic Th17 cells and displayed metabolic syndrome and NASH. Notably, administration of anti-IL-17A antibody ameliorated hepatic inflammation but not affecting steatosis and liver damage [20]. Inhibition of lipogenesis by suppressing acetyl-CoA carboxylase 1 and 2 inhibited Th17 cell differentiation by naïve CD4^+^ T cells [29], suggesting the critical role of lipogenesis in controlling Th17 cell polarization. Collectively, these findings highlight shaping of Th17 differentiation by lipid metabolic changes and the critical role of activated Th17 cells in the progression of NAFLD to NASH.

## 3. Effects of Maternal Nutrition on the Development of Offspring NAFLD

Exposure to maternal malnutrition in utero plays a pivotal role in the later-life development of NAFLD in offspring. In a mouse model, Purcell et al. demonstrated that intervening in maternal weight during pregnancy reduces hepatic inflammation and improves hepatic health, thereby preventing the onset of metabolic-associated fatty liver disease in offspring [30]. In humans, the quality of maternal diet during gestation is associated with hepatic lipid deposition in early offspring life [31]. Additionally, maternal exposure to a high-fat diet during gestation and lactation exacerbates NAFLD phenotypes in offspring fed a high-fat diet post-weaning. Interestingly, maternal supplementation with one-carbon corrects these outcomes [32]. A meta-analysis of datasets also shows that maternal metabolic dysfunction and diet during pregnancy are early developmental factors associated with offspring NAFLD [33]. Taken together, these findings indicate that maternal nutrition programs the pathogenesis of NAFLD in the early life of offspring.

### 3.1. Hepatic Lipogenesis

Indeed, de novo fatty acid synthesis in the fetal liver is crucial for fetal development, as a deficiency in fatty acid synthase results in embryo demise in utero [34]. However, higher activity of de novo hepatic lipogenesis and elevated triglyceride deposition in the liver are critical pathogenic features in patients with NAFLD. A study utilizing nonhuman primates demonstrated that maternal consumption of an obesogenic diet before and during pregnancy led to increased hepatic oxidative stress and induced steatotic liver disease in offspring [35]. Moreover, another nonhuman primate study revealed that offspring born to mothers with a high-fat diet and insulin resistance exhibited upregulation of genes involved in de novo lipogenesis in their livers, along with increased hepatic inflammation characterized by enhanced infiltration of alternatively activated hepatic macrophages and natural killer T cells. Intriguingly, these hepatic phenotypic changes in offspring were found to be irreversible upon switching to a control diet [36]. These results suggest that maternal diet-associated metabolic alterations program the de novo lipogenic pathways and dysregulate the hepatic immune system in offspring. Furthermore, mitochondrial dysfunction leading to increased reactive oxygen species (ROS) generation also plays a critical role in NAFLD pathogenesis. Hepatic mitochondrial dysfunction has been associated with heightened oxidative stress and impaired respiratory capacity [37]. Maternal consumption of a Western-style diet has been shown to affect the hepatic mitochondrial respiratory chain complex in offspring, thereby promoting NAFLD development postnatally. These effects include reduced activity of mitochondrial respiratory chain complexes [38], inhibition of cytochrome c expression [39], and decreased hepatic mitochondrial DNA copy number [40]. Additionally, multiple factors, including gut dysbiosis, epigenetic modifications, and inflammatory responses influenced by maternal overnutrition, also increase the risk of offspring developing NAFLD (refer to Figure 1).

### 3.2. Insulin Resistance

Insulin resistance is frequently linked to the development and progression of NAFLD. Under physiological conditions, insulin engagement with its receptor activates SREBP1, promoting hepatic de novo lipogenesis. However, in insulin-resistant states, often associated with hyperinsulinemia, this pathway is amplified, leading to the accumulation of large amounts of free fatty acids in the liver and facilitating NAFLD progression [41]. Maternal overnutrition during pregnancy has been reported as a risk factor for offspring developing obesity and NAFLD in adulthood. Indeed, in rat models, maternal consumption of a high-fat diet during gestation and lactation resulted in decreased mass of insulin-producing β-cells [42]. This led to a compensatory increase in insulin secretion, resulting in hyperinsulinemia. In contrast, exposure to a high-fat intrauterine environment increased pancreatic α-cell mass, the major source of glucagon, leading to hyperglycemia [43]. As hyperglycemia and hyperinsulinemia are critical factors contributing to hepatic lipogenesis through the activation of ChREBP and SREBP-1, respectively [41], maternal overnutrition-induced alterations in pancreatic function appear to link to NAFLD programming in offspring. In contrast, studies have shown that maternal under-nutrition during pregnancy results in reduced pancreatic β-cell mass and impaired β-cell differentiation [44]. These alterations lead to reduced insulin secretion, prompting compensatory elevation in insulin production and subsequent hyperinsulinemia [45]. Thus, exposure to nutritional stress in utero programs pancreatic development, potentially linking to NAFLD development in adulthood.

### 3.3. Maternal Under-Nutrition

A low birth weight due to intrauterine growth restriction (IUGR), stemming from maternal under-nutrition, also poses a risk for offspring obesity and metabolic syndrome. IUGR can induce placental dysfunction, diminishing placental nutritional transport to the fetus, resulting in abnormal fetal growth and adverse outcomes [46]. Studies have demonstrated that IUGR triggers an abnormal developmental leptin surge, leading to increased production of orexigenic neuropeptides and hyperphagia, thereby promoting fat deposition in adipose tissue [45]. In the context of NAFLD, rat models subjected to prenatal food restriction exhibited reduced expression of *Pparα* and *Pparγ*, alongside elevated levels of *Srebp1c* and *Fasn* in the liver [47]. This prenatal food restriction was associated with hepatic steatosis in late adulthood when exposed to a high-fat diet post-weaning [48]. Human studies have confirmed a significant U-shaped relationship between birth weight and NAFLD [49], indicating that both low and high birth weights elevate the risk of NAFLD development in children. However, investigations into the contributions of factors such as gut microbiota and epigenetic alterations to offspring NAFLD induced by maternal under-nutrition remain scarce.

## 4. Interplay of Early-Life Nutrition, Gut Microbiome, and Hepatic Inflammation

### 4.1. Gut Microbiome in Early Life

In recent years, accumulating evidence indicates that nutrition plays a pivotal role in shaping the composition of the gut microbiome and its metabolites. These factors subsequently contribute to the development and regulation of the immune system [50]. The regulation of immune activation by the gut microbiome determines its influence on host health and disease. Additionally, it has been demonstrated that transmission of maternal intestinal bacteria to the neonate shapes colonization patterns in the neonatal gut during the gestation and lactation periods [51]. Indeed, while the gut microbiome of infants is highly variable, it begins to more closely resemble that of their mothers as they grow older [52,53], suggesting the impact of maternal diet on the gut microbiome and immune system development in offspring. Indeed, in a non-human primate model, microbial dysbiosis in offspring was induced by maternal high-fat diet and was only partially reversible by feeding a low-fat diet after weaning [54], indicating that maternal diet contributes to the establishment of newborns’ microbial communities. Fetuses acquire microbial colonization derived from the maternal birth canal and expand with high diversity in early life [55]. The composition of gut microbiota in a healthy state typically comprises 6 phyla, with Firmicutes and Bacteroidetes being the predominant species [56]. These microbial communities play a crucial role in maintaining homeostasis. Alterations in microbial composition, such as the depletion of specific bacterial species, disrupt the balance of interactions among bacterial species, ultimately contributing to disease. Imbalances in bacterial communities in infants may increase the risk of several diseases, such as allergies and autoimmune diseases, later in life [57]. Notably, germ-free mice transplanted with fecal microbes from infants exposed to obese mothers were predisposed to NAFLD. This was accompanied by an increase in intestinal permeability and the hepatic expression of pro-inflammatory cytokine genes, including *Tnfα*, *Ifnb1*, and *Il6*. Additionally, these mice exhibited increased periportal leukocyte infiltration, suggesting hepatic inflammation [58].

### 4.2. Microbial Composition

Certain microbial organisms in the intestines of offspring have been implicated in shaping the immune response. For instance, maternal intake of human milk-derived *Lactiplantibacillus plantarum* during lactation increased the levels of intestinal Bacteroides in offspring early in life, along with elevated serum levels of IL-6 and increased splenic CD4^+^ T lymphocytes [59]. Newborn mice from mothers supplemented with butyrate showed an enrichment of fecal microbial signatures of *Bacteroidetes* and *Clostridia*, which are linked to the prevention of hepatic immune cell activation [60]. Additionally, *Bacteroides* can produce sphingolipids that are transported to the liver to enhance β-oxidation, resulting in reduced lipid accumulation [61]. Maternal nutrition influences the composition of the gut microbiota in offspring born to mothers with overnutrition. Folic acid supplementation during pregnancy alters the abundance of *Desulfobacterota* and the *Firmicutes*/*Bacteroidota* (F/B) ratio in male offspring, which alleviates hepatic lipid accumulation and inflammatory responses in offspring of high-fat diet-fed dams [62].

### 4.3. Gut-Liver Axis

The influence of NAFLD pathogenesis by intestinal microbiome-associated factors is attributed to the connection between the gut and liver through the portal vein and biliary duct [63]. Disruption of the intestinal barrier due to diet-induced alterations in gut microbial balance facilitates the influx of microbiome-associated derivatives, such as lipopolysaccharide (LPS) and metabolites, into the liver, thereby exacerbating hepatic inflammation [64]. Indeed, intestinal epithelial barrier function plays a crucial role in the progression of hepatic steatosis. Mice with genetic deletion of the *F11r* gene, which encodes junctional adhesion molecule A (JAM-A), a protein that regulates tight junctions, exhibit defects in the maintenance of intestinal permeability and develop severe steatohepatitis on a Western-style diet compared to wild-type mice. Moreover, treatment of *F11r*-ablated mice with antibiotics reduces Western diet-induced steatohepatitis [65]. Furthermore, neonatal exposure to the gut microbiome from wild mice prevents obesity and hepatic steatosis [66]. Interestingly, these wild microbiome-associated phenotypes occur only with early-life exposure and not in adulthood, suggesting the key role of the gut microbiome in metabolic programming in early life [66].

### 4.4. Gut Bacteria and NAFLD Pathogenesis

Emerging evidence implicates a transgenerational impact on gut microbial flora and its connection to liver disease. Transplantation of germ-free mice with fecal samples from infants of obese mothers predisposes them to the development of pediatric NAFLD, underscoring the direct contribution of gut microbial compositions to NAFLD pathogenesis [58]. Transplantation of gut bacteria harvested from mothers on a high-calorie diet into germ-free mice resulted in fasting hyperglycemia and increased hepatic fat deposition [67]. Furthermore, dysbiosis in offspring born to mothers with overnutrition increases gut permeability and myeloid cell infiltration of the liver, leading to developmental programming of NAFLD [68], suggesting that maternal diets predispose offspring to NAFLD through the regulation of gut microbial communities. Indeed, disturbance of the gut microbial ecosystem by the uptake of probiotic *Lactobacillus reuteri* during gestation prevents maternal high-fat diet-induced fetal liver steatosis [69]. Collectively, these results suggest that microbial dysbiosis contributes to the onset of hepatic inflammation.

### 4.5. Metabolites of Gut Bacteria and NAFLD Pathogenesis

Gut dysbiosis induces impaired intestinal barrier integrity, leading to the translocation of bacteria-derived substances to the liver and modulation of hepatic lipid metabolism and inflammation. For instance, intestinal bacteria produce short-chain fatty acids (SCFAs) such as acetate, propionate, and butyrate via anaerobic fermentation, which stimulate the intestinal epithelium for nutrient sensing and energy balance. Additionally, intestinal microbiota-derived SCFAs play a critical role in the development of NAFLD. The effects of SCFAs on the regulation of NAFLD pathogenesis can be transmitted via maternal nutrition to offspring. Indeed, maternal betaine intake modulates the gut microbiome ecosystem, resulting in a decrease in *Proteobacteria*, *Desulfovibrio*, and *Ruminococcus* strains, accompanied by an increase in *Bacteroides* and *Parabacteroides*. These changes increase the fecal concentrations of microbial SCFAs, including acetic acid, butyric acid, and valeric acid, correlating with increased expression of genes involved in the suppression of hepatic lipid metabolism such as *Pparα*, *Cpt1α*, and *Fatp2*. This, in turn, alleviates the maternal high-fat diet-induced development of NAFLD in offspring mice [70]. In contrast, maternal sucralose administration disturbs the gut microbial composition by reducing butyrate-producing bacteria in offspring, resulting in decreased cecal butyrate levels. This leads to the attenuated anti-inflammatory effect of butyrate through the downregulation of GPR43, thereby facilitating hepatic steatosis in adulthood when exposed to a high-fat diet [71]. Oral supplementation of butyrate to gestated rats prevents maternal high-fat uptake-induced fetal liver oxidative injury and steatosis [69]. The suppressive effects of butyrate on NAFLD progression are, at least partly, attributed to its regulatory role on microRNA (miRNA). Mice treated with butyrate upregulate the level of miR-150, which targets the chemokine receptor CXCR4, thereby reducing hepatic inflammatory response and attenuating NAFLD progression [72]. Additionally, acetate and butyrate have been reported to regulate the miR-378a/YY1 axis and exhibit a beneficial effect on metabolic disorders [73].

### 4.6. Bile Acids

Bile acids play a crucial role in gut-liver interactions. Gut microorganisms can convert liver-secreted primary bile acids to secondary bile acids and promote their drainage into the liver via portal circulation [74]. Patients with NAFLD often exhibit increased serum levels of secondary bile acids but reduced hepatic bile acid signaling [75]. In a murine model, stimulation of the bile acid receptor farnesoid X receptor (FXR) in the intestines by fexaramine has been shown to reduce diet-induced liver metabolic dysfunctions [76]. However, deletion of intestinal FXR signaling in mice reduces the progression of NAFLD induced by a high-fat diet [77], suggesting a promotive role of bile acids in the development of NAFLD. Nonetheless, secondary bile acids have been reported to modulate the balance between Th17 and Treg cells, which possess immunostimulatory and immunosuppressive activities, respectively, by directly binding to the master transcription factor retinoid-related orphan receptor-γt to inhibit Th17 differentiation and stimulate mitochondrial reactive oxygen species to promote Treg differentiation [78]. Maternal high-calorie diet also impacts offspring bile acid pool. Female mice fed with a high-fat/high-sucrose diet can increase the intrahepatic bile acid pool size and decrease secondary bile acid production, which is associated with worse hepatic steatosis in offspring exposed to Western-style diets [79].

## 5. Effects of Maternal Nutrition on Epigenetic Remodeling in Offspring

### 5.1. DNA Methylation

Epigenetic alterations, including DNA methylation, histone modification, and microRNA (miRNA) activity, play pivotal roles in regulating immune-metabolic characteristics and metabolic disorders such as NAFLD. Genome-wide association studies (GWASs) have demonstrated that genetic variants affecting gene expression and/or activity in sugar and lipid metabolic pathways increase the incidence of hepatic steatosis [80]. Moreover, accumulating evidence implicates epigenetic factors in NAFLD development. Histone modification changes are reported to “memorize” intrauterine exposure to a high-fat diet, potentially contributing to liver disease development in adulthood [81]. This underscores the critical role of maternal nutrition in offspring’s metabolic liver disease development through epigenetic regulation. For instance, mice fed a high-fat diet exhibited altered DNA methylation patterns in metabolism-related genes in their female offspring livers, though not in imprinted genes [82]. Intriguingly, a recent study in a mouse model revealed that offspring exposed to adverse early-life environments, including maternal Western diet, displayed altered hepatic DNA methylome prior to transcriptome changes. The differentially expressed genes were enriched for liver metabolic functions and correlated with the onset of NAFLD later in life [83]. Furthermore, maternal high-fructose diet induced sustained changes in DNA methylation and gene expression in the liver stem/progenitor cells of offspring. Functional analysis revealed that the affected genes were enriched for metabolic processes and ion transport [84]. These findings suggest the impact of maternal overnutrition on offspring NAFLD development through modulation of DNA methylation levels in genes involved in hepatic metabolic processes. Indeed, recent reports indicate that male offspring born to mothers with hypercholesterolemia are predisposed to NAFLD. Mice fed a Western-style diet during pregnancy and lactation induced hepatic steatosis in their offspring at 4 months of age, even though the offspring were weaned onto a regular diet postnatally. The hepatic methylation of male offspring showed an increase in the promoter region of the *ApoB* gene, reducing the ApoB level in serum and liver, thus contributing to hepatic steatosis [85]. Additionally, maternal exposure to a high-fat diet or one-carbon supplement induces differential methylation patterns in genes associated with phospholipid metabolism, such as *Prkca*, *Dgkh*, *Plcb1*, and *Dgki*, in offspring and subsequent development of hepatic steatosis [32].

Human studies have also revealed alterations in hepatic DNA methylation profiles between patients with NAFLD and healthy subjects. Patients with NAFLD have been observed to exhibit lower hepatic global DNA methylation compared to control groups. This reduction in hepatic DNA methylation patterns tends to be associated with increased hepatic inflammation and disease progression [86]. Another study demonstrated that differentially expressed hepatic DNA methylation patterns can distinguish patients with advanced or mild NAFLD [87]. In a cohort study involving 40 mother-newborn pairs, maternal lipid metabolites were found to be correlated with cord blood metabolites. Notably, these metabolites were associated with fetal DNA methylation states [88].

### 5.2. microRNA and Histone Modifications

Other epigenetic modifications affecting NAFLD pathogenesis include histone modification and microRNA (miRNA)-mediated gene expression. In a genetically engineered mouse model of maternal high-fat diet during pregnancy, disruption of the C/EBPα and histone acetyltransferase p300 interaction by substituting serine with alanine at residue 193 on C/EBPα prevents pups from developing NAFLD compared to wildtype mouse pups. Accordingly, the C/EBPα/p300 complex-induced expression of genes involved in NAFLD progression is blocked in mutant mouse pups [89]. In nonhuman primates, Suter et al. demonstrated that alterations in fetal acetylation of histone H3 (H3K14ac) were associated with maternal high-fat diet-induced NAFLD in offspring. Furthermore, they unveiled decreased expression of SIRT-1 and its deacetylase activity in offspring exposed to a high-fat diet in utero. This resulted in an increased level of H3K14ac in fetal livers, accompanied by the upregulation of SIRT-1-suppressed genes involved in lipid metabolism, such as *PPARA*, *PPARG*, *SREBF1*, *CYP7A1*, *FASN*, and *SCD* [90]. Transgenerational high-fat diet feeding downregulates G9a, a histone methyltransferase acting on H3K9 dimethylation, and reduces methylated histones on the gene promoters of *LXRα* and *ERO1-α*, leading to the activation of lipogenesis in offspring livers [91].

Several circulating miRNAs, including miR-122, miR-34a, miR-15b, and miR-16, have been identified in patients with NAFLD, with their upregulation positively correlated with NAFLD pathogenesis [92]. Interestingly, an inverse correlation has been observed between circulatory and liver miRNA levels. Among these, miRNA-122 is the most abundant hepatic miRNA, playing a pivotal role in lipid metabolism. Downregulation of miR-122 in livers increases the expression of its target genes, which are implicated in lipid metabolism and predict human NASH [93]. Alterations in hepatic miRNAs have also been observed in offspring of mothers fed a high-fat diet during gestation and lactation [94]. The expression of miR-122 in offspring from maternal high-fat feeding was reduced. Moreover, deletion of miRNA-122 resulted in hepatic steatosis and inflammation [95]. These findings collectively suggest that epigenetic influences contribute to NAFLD pathogenesis, and these epigenetic programming can be transgenerationally transmitted due to early-life malnutrition.

## 6. Impacts of Maternal Diet-Induced Epigenetic Remodeling on Immune Regulation

Epigenetic modifications affecting immune functions in the context of NAFLD have been explored [96]. Emerging evidence has also shown a link between maternal obesogenic factors and epigenetic alterations in fetal immune cell reprogramming [97]. These changes regulate the interplay between environmental insults and genetics to induce the inflammatory responses commonly seen in NAFLD. For instance, there is an increase in CD4^+^ T cell frequency and a decrease in myeloid populations in cord blood mononuclear cells from newborns of obese mothers [97]. Furthermore, umbilical cord blood obtained from offspring born to mothers with obesity exhibits an increase in various lymphocyte subsets, including CD3^+^ T cells, natural killer T cells, and CD8^+^ regulatory T cells [98]. Additionally, newborns from obese mothers show altered cytokine profiling in cord blood monocyte-differentiated macrophages, and they display a differential response to M2 type stimulation. These changes in cytokine expression are associated with DNA methylation status in the promoter region of genes controlling these cytokines’ transcription in fetal monocytes [99]. Together, these results suggest that maternal nutrition reprograms offspring immune function through epigenetic regulation in utero.

Indeed, fetal macaques exposed to a maternal high-fat diet show impaired differentiation of fetal hematopoietic stem/progenitor cells (HSPCs), characterized by the induction of a proinflammatory response in fetal macrophages and suppression of genes involved in B cell development. These offspring also exhibit reduced B cell frequency compared to those born to mothers on a regular diet [100]. Another report demonstrates that fetal liver HSPCs display pro-inflammatory phenotypes with intrauterine exposure to a Western-style diet in nonhuman primate models [101]. Furthermore, similar phenotypes are observed in bone marrow-derived macrophages (BMDMs) and bone marrow HSPCs from 3-year-old juvenile offspring, suggesting that the in-utero transmission of maternal diet-induced long-term pro-inflammatory memory in innate immune cells occurs through HSPCs. Perturbations of fetal HSPCs by inflammatory stimuli from maternal diet interfere with the immune profiling and function of fetal immune cells. Conversely, the intrauterine memory of innate immune cell response to inflammatory stimuli (e.g., maternal diet or infections) may be stored in adult hematopoietic stem cells (HSCs) through epigenetic modifications, providing direct sensitivity and responsiveness to secondary insults across the lifespan [102]. These findings highlight that maternal nutrition susceptible to NAFLD induction modulates the immune cell developmental programming in fetal HSPCs.

While the impact of T cells on the progression of hepatic steatosis to steatohepatitis and hepatocarcinogenesis has been unveiled [13], the influence of fetal HSPCs exposed to maternal nutrition on T cell phenotypes and their subsequent contribution to the development of NAFLD in adulthood requires further investigation. Among the T cell populations in fetal liver, regulatory T cells (Tregs) are recognized as key players in immunological tolerance due to their anti-inflammatory capacity. Thus, in the fetus, to prevent mounting an immune response against the mother, a higher number of Tregs derived from fetal HSPCs than adult HSPCs leads to bias toward immune tolerance [103]. It would be valuable to investigate whether changes in Tregs frequency in the fetal liver exposed to maternal malnutrition.

## 7. Therapeutic Intervention

**Figure 2:**
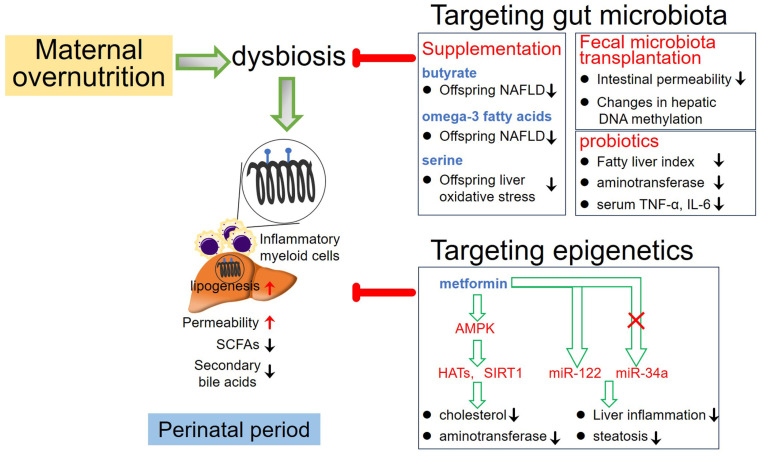
Potential therapeutic interventions aimed at mitigating the risk factors induced by maternal overnutrition and influencing the development of offspring NAFLD. SCFAs: short-chain fatty acids; HATs: histone acetyltransferase.

Manipulation of gut microbial composition through fecal microbiota transplantation (FMT), probiotics, and antibiotics has emerged as therapeutic strategies for treating liver disease, showing promising clinical outcomes. Studies have demonstrated that allogeneic FMT delivery to NAFLD patients reduced small intestinal permeability, whereas autologous FMT delivery showed no significant changes in gut barrier integrity [104]. Furthermore, NAFLD patients receiving allogeneic FMT intervention exhibited changes in gut microbiota composition, plasma metabolites, and hepatic DNA methylation patterns compared to those receiving autologous FMT [105]. This suggests potential therapeutic effects on NAFLD patients. Additionally, introducing stool from healthy donors enriched in specific microbial species has shown to restore antibiotics-induced reduction of microbial diversity and impairment of gut microbial function in patients with advanced cirrhosis [106], indicating the significant contribution of specific gut microbial species to the management of liver disease. In fact, treatment with probiotics has also shown promise in NAFLD management. For instance, NAFLD patients treated with a probiotic mixture containing six bacterial species experienced reductions in body weight and intrahepatic fat fraction [107]. Similarly, a multi-probiotic formula including 14 probiotic bacteria genera exhibited therapeutic efficacy on NAFLD patients, resulting in reduced fatty liver index, aminotransferase activity, and serum levels of TNF-α and IL-6 [108]. Figure 2 outlines potential therapeutic interventions that could mitigate the risk factors induced by maternal overnutrition and influence the development of offspring NAFLD.

Oral supplementation of propionate, a short-chain fatty acid (SCFA) derived from gut microbiota, has been shown to prevent the increase in intrahepatic lipid compared to control groups [109], indicating potential improvement of NAFLD by gut bacterial metabolites. Furthermore, in a population-based cohort study of 2424 mother-child pairs, diet intervention involving maternal uptake of omega-3 polyunsaturated fatty acids during gestation was associated with lower childhood liver fat and reduced risk of offspring NAFLD development in childhood [110]. A preclinical study has also indicated the potential of maternal gut microbiota-targeted therapy, such as the use of probiotic *Lactobacillus reuteri* or the metabolite butyrate, to ameliorate the programming of NAFLD induced by postnatal high-fat diet exposure [69]. However, further research is needed to define the clinical benefits of modulating gut microbial composition on NAFLD via transgenerational transmission from mothers to their offspring.

Emerging evidence suggests the therapeutic potential of correcting dysregulated epigenetic modifications in NAFLD. Analysis of DNA methylation patterns in the liver reveals significant differences between patients with NAFLD and healthy subjects, which are associated with the severity of hepatic inflammation and disease progression, providing a basis for targeting epigenetic alterations in NAFLD treatment. Modulation of gene expression to regulate metabolic pathways by targeting miRNA may be a promising strategy for treating metabolic diseases [111]. Preclinical study has demonstrated that targeting miR-34a in obese mice normalized SIRT1 activity, leading to the deacetylation of regulatory genes involved in hepatic lipid metabolism, accelerated β-oxidation, and suppressed lipogenesis, thereby reducing hepatic steatosis [111]. In clinical investigations, the implication of resveratrol and GLP-1 (Glucagon-like peptide-1) receptor agonists, both of which can activate SIRT1, in NAFLD treatment has been investigated. Clinical meta-analyses have shown that resveratrol intake ameliorated NAFLD primarily through AMPK/SIRT1 and anti-inflammatory pathways [112]. The GLP-1 receptor agonist semaglutide improves several pathological characteristics of NAFLD, including reduced steatosis, lobular inflammation, hepatocellular ballooning, and alleviation of NASH progression [113]. Additionally, liraglutide treatment results in histological resolution in a portion of patients with NASH [114].

Despite promising efficacy of some dietary agents targeting DNA methylation or histone modifications in improving NAFLD in preclinical studies, their clinical translational relevance is still lacking. However, some existing drugs have demonstrated the potential for epigenetic modulation. For example, metformin, a drug used for type 2 diabetes, influences a panel of epigenetic-modifying enzymes, particularly mediating the activation of AMPK. Activated AMPK by metformin enhances histone acetyltransferase (HATs) activity and increases HDAC SIRT1 activity, causing DNA hypermethylation at the promoter of genes involved in metabolic pathways [115]. These effects suggest metformin as a promising drug for treating NAFLD by targeting epigenetic modifications. Preclinical studies have shown that metformin treatment of rat dams fed with a high-fat diet alleviates hepatic steatosis in early adulthood of offspring by reducing placental oxidative stress and elevating glucose transporter 1 (GLUT1), GLUT3, and GLUT4 expression in the placenta [116].

As aforementioned, fetal HSPCs store the “memory” of environmental perturbations in utero via epigenetic modifications to adult HSPCs, and the changes in fetal HSPCs can manifest in their myeloid progeny cells. However, the effects of epigenetic shifts on lymphoid progeny cells remain unclear, despite no changes in T cell frequency in fetal bone marrow cells exposed to maternal Western-type diet in utero [101]. Interestingly, the short-chain fatty acid butyrate produced by gut microbiota programs macrophages to an antimicrobial state by impairing histone deacetylase 3 (HDAC3) [117]. Additionally, both butyrate and propionate promote the generation of peripheral regulatory T cells by HDAC inhibition [118].

## 8. Conclusions

In the context of NAFLD, fetal exposure to maternal malnutrition induces alterations in gut microbial flora and epigenetic programming that regulate the expression of genes involved in lipid metabolism and the immune response. Understanding the interaction between the mother-fetus interface, particularly the transmission of phenotypes from maternal diet, and its long-term influence on the gut microbiome and epigenetic network is conducive to developing strategies to prevent or treat NAFLD. Additionally, the identification of new biomarkers for monitoring NAFLD progression through genetic and epigenetic approaches can provide early detection of an individual’s susceptibility to NAFLD.

## Data Availability

Not applicable.
